# Lewis Phenotype in Women With Preterm Labor and Premature Rupture of the Membranes

**DOI:** 10.1155/S1064744995000329

**Published:** 1995

**Authors:** William F. O'Brien, German F. Leparc, Jodi Holbrook

**Affiliations:** Department of Obstetrics and Gynecology University of South Florida College of Medicine and Southwest Florida Blood Bank 4 Columbia Drive, #506 Tampa FL 33606 USA

## Abstract

*Objective:* The purpose of this study was to evaluate the possible association between Lewis phenotype status in pregnant women and preterm labor (PTL) or preterm rupture of the membranes (PROM).

*Methods:* Red blood cell (RBC) Lewis phenotype was determined in 113 pregnant women admitted for PTL or PROM and in 121 controls. The results were controlled for the influence of race on Lewis phenotype.

*Results:* Pregnancy was associated with a higher frequency in women with the a–b– phenotype. There was no association between RBC Lewis phenotype and the occurrence of PTL or PROM.

*Conclusions:* A susceptibility to PTL or PROM is not due to a lack of Lewis antigen expression on the plasma membrane of the vaginal mucosa.


Infectious Diseases in Obstetrics and Gynecology 3:60–63 (1995)
© 1995 Wiley-Liss, Inc.

### Lewis Phenotype in Women With Preterm Labor and Premature Rupture of the Membranes

**William F. O'Brien, German F. Leparc, and Jodi Holbrook**

*Department of Obstetrics and Gynecology, University of South Florida College of Medicine and Southwest Florida Blood Bank, Tampa, FL*

ABSTRACT

***Objective:*** **The purpose of this study was to evaluate the possible association between Lewis phenotype status in pregnant women and preterm labor (PTL) or preterm rupture of the membranes (PROM).**

***Methods:*** **Red blood cell (RBC) Lewis phenotype was determined in 113 pregnant women admitted for PTL or PROM and in 121 controls. The results were controlled for the influence of race on Lewis phenotype.**

***Results:*** **Pregnancy was associated with a higher frequency in women with the a−b− phenotype. There was no association between RBC Lewis phenotype and the occurrence of PTL or PROM.**

***Conclusions:*** **A susceptibility to PTL or PROM is not due to a lack of Lewis antigen expression on the plasma membrane of the vaginal mucosa.** © 1995 Wiley-Liss, Inc.

KEY WORDS

Lewis antigen, infection, pregnancy, race

Lewis antigens are a group of oligosaccharides found in red blood cells (RBCs), plasma, saliva, and vaginal and urinary-tract epithelium. These fucosylated carbohydrate chains, which are secreted by a variety of cell types, become passively incorporated into the cell membrane of RBCs through adsorption from the plasma. Individuals who lack the Lewis genotype and fail to express these antigens are typed as Le a−b−. Those who possess the Lewis genotype but do not express secretor status (se/se) have a variant form of Lewis antigen expression typed as a+b−. Most people have both Lewis and secretor expression. The individuals are typed as a+b+.

Recent studies have demonstrated an association between the expression of Lewis antigens on urothelial plasma membranes and a resistance to urinary-tract infection.[1,2]

In view of the current interest in the possible role of microbial invasion of the uterus in premature birth, we sought to investigate the possible association between these complications and Lewis antigen status in a group of pregnant women. Specifically, we sought to determine whether the absence of Lewis antigen expression occurs more frequently in women with pregnancies complicated by preterm labor (PTL) or premature rupture of the membranes (PROM).

### SUBJECTS AND METHODS

Samples were obtained from women with singleton pregnancies presenting for care at the Tampa General Hospital. Venous blood samples obtained for the determination of blood type and antibody status were utilized for the determination of Lewis antigen expression on the RBCs.

Samples from 3 groups of women were selected. The control subjects were women presenting in labor at term (>37 weeks gestational age) with

Address correspondence/reprint requests to Dr. William F. O'Brien, Department of Obstetrics and Gynecology, University of South Florida College of Medicine, 4 Columbia Drive, #506, Tampa, FL 33606.

Received March 16, 1995

Accepted July 5, 1995

Clinical Study

**TABLE 1. Clinical characteristics of the study populations[a]**

| | Control (N = 121) | PTL (N = 47) | PROM (N = 66) | P* |
|---|---|---|---|---|
| Age | 22.3 ± 5.8 | 21.3 ± 0.8 | 23.5 ± 0.7 | 0.36 |
| EGA-ADM | 39.7 ± 0.3 | 32.4 ± 0.4 | 30.8 ± 0.6 | <0.001 |
| EGA-DEL | 40.1 ± 0.4 | 34.9 ± 0.6 | 32.6 ± 1.0 | <0.001 |
| Birth weight | 3,307 ± 34 | 2,449 ± 109 | 1,836 ± 101 | <0.001 |
| White N (%) | 66 (54) | 20 (42) | 30 (45) | 0.28 |

[a]EGA-ADM = estimated gestational age at admission; EGA-DEL = estimated gestational age at delivery.
*P = comparison between PTL and PROM groups to control.

intact membranes following an uncomplicated antenatal course. The PTL group consisted of women with intact membranes presenting with idiopathic PTL (<37 weeks gestational age). All women in this group had evidence of PTL documented by frequent uterine contractions (5 or more contractions in a 20-min period) and documented progressive cervical dilation. The PROM group consisted of women who presented for care with spontaneous rupture of the membranes (gestational age earlier than 38 weeks) prior to the onset of uterine contractions.

The laboratory personnel responsible for determining the phenotype had no knowledge of the patient's medical history. The venous samples were placed in sterile test tubes without anticoagulant. All tests were completed within 24 h of sampling. A 3–5% saline suspension of one-time-washed RBCs was prepared using isotonic saline. The Lewis phenotyping was performed by incubating 1 drop each of anti-Lea or anti-Leb murine monoclonal blood grouping reagent (Bioclone, Ortho Diagnostic Systems, Raritan, NJ) into the respective tubes. Following an incubation for 10 min at room temperature, the tubes were centrifuged at 3,400 rpm for 20 seconds. Following a manual resuspension by gentle agitation, the macroscopic hemagglutination was assessed. If a reaction was seen with both anti-Lea and anti-Leb reagents, a direct antiglobulin test was performed to determine whether the reactivity was due to the presence of IgG on the RBC surface.

Statistical calculations were performed with the SAS analysis package using the corrected chi-squared test. A $P < 0.05$ probability level was used as a measure of significance. The power analysis assumptions included an estimate of the Le (a−b+) phenotype in the control population, an increase in incidence to 75% in the PTL and PROM populations, an $\alpha$ of 0.05, and a power of 80%. These estimates yielded a required sample size of 98 subjects in each group.

### RESULTS

The clinical characteristics of the study groups are presented in Table 1. As expected, the PTL and PROM groups had significantly shorter pregnancy durations and lower birth weights. The frequency of whites was nonsignificantly higher in the control group, with similar frequency in the PTL and PROM groups.

The Lewis phenotypes determined in our pregnant population were at a considerable variance from those obtained in our nonpregnant population.[3,4] As evident in Table 2, both black and white populations had a higher than expected frequency of Le (a−b−) phenotype determination. This higher frequency is most likely due to the effect of pregnancy on the determination of the Lewis phenotype with false assignment of the Le (a−b−) phenotype.[5]

A comparison of the Lewis phenotype determination between the control group and the PTL or PROM group may be seen in Table 3. The relative proportions of Lewis phenotype in both black and white populations were almost identical when the control group and PTL or PROM group were compared.

### DISCUSSION

The association between an increased risk for urinary-tract infection and the absence of Lewis antigens is assumed to be secondary to the protection from bacterial attachment afforded by fucosylated carbohydrate chains expressed on the urogenital cells of women who possess Lewis antigens. Bacteriuria

**TABLE 2. Effect of pregnancy on Lewis phenotype determination according to race**

| | White | | Black | |
|---|---|---|---|---|
| | Observed | Nonpregnant | Observed | Nonpregnant |
| a−b+ | 71 (61%) | (73%)* | 51 (43%) | (51%)* |
| a+b− | 26 (22%) | (20%) | 18 (15%) | (20%) |
| a−b− | 19 (16%)* | (6%)** | 49 (42%)* | (29%)** |

*$P < 0.001$, $\chi^2$; black vs. white.
**$P < 0.01$, $\chi^2$; pregnant vs. nonpregnant.

**TABLE 3. Lewis phenotype in women with PTL or PROM**

| | Control | PTL | P |
|---|---|---|---|
| | | *White* | |
| a−b+ | 38 (57%) | 33 (66%) | 0.65 |
| a+b− | 16 (24%) | 10 (20%) | |
| a−b− | 12 (18%) | 7 (14%) | |
| | | *Black* | |
| a−b+ | 21 (38%) | 30 (48%) | 0.58 |
| a+b− | 9 (16%) | 9 (14%) | |
| a−b− | 25 (46%) | 24 (38%) | |

in women is preceded by an attachment of pathogenic, coliform, gram-negative organisms to cells lining the vaginal introitus. This attachment is dependent upon the pathogenic bacteria's expression of pili which facilitate attachment to the cells of the vaginal epithelium. This attachment is inhibited in women who express Lewis antigens through interference in contact between the pili and the epithelial cell membrane.

The expression of Lewis antigens on the cell membrane depends upon an interaction between the Lewis genotype and the secretor locus. Individuals who possess both Lewis and secretor genes are phenotypically recognized as Le (a+b+). The presence of the Lewis gene with the absence of the secretor gene activity yields a Le (a+b−) phenotype. The absence of the Lewis gene yields a Le (a−b−) phenotype regardless of secretor status. Recent studies utilizing immunohistological staining with monoclonal antibodies have confirmed the expression of these antigens in vaginal epithelial cells and mucus.[6] Since the maximal expression of Lewis carbohydrate chains on cell membranes is associated with the Le (a+b+) phenotype, women with either Le (a+b−) or Le (a−b−) would be expected to be at an increased risk for bacterial attachment to the vaginal epithelium.

The association between Lewis antigen status and urinary-tract infection has been documented in children with structural anomalies of the urinary tract[1] and in women with recurrent urinary-tract infection.[2]

Several lines of investigation have implicated bacterial contamination of the amniotic cavity as an important component in PTL and PROM. Colonization of the amniotic cavity with organisms associated with the vaginal flora has been a consistent finding in studies of amniocentesis specimens from women with PTL and PROM.[7,8] Although the percentage of women colonized and the organisms cultured vary among these studies, the subclinical infection is clearly an important cause of these pregnancy complications in some of these women. A pathogenic mechanism for this association, moreover, has been advanced with the finding of increased levels of cytokines in colonized samples. These cytokines, particularly the interleukins, can result in an increase in amnion-cell prostaglandin production, resulting in myometrial contractions and subsequent labor.[9,10]

In our study, we confirmed the previously documented altered distribution of erythrocyte Lewis phenotype expression during pregnancy. In both white and black populations, there was a higher than expected incidence of Le (a−b−) women and a correspondingly lower than expected frequency of Le (a+b+) phenotypes. This apparent change in distribution is most likely due to a reduction in serum transferase[4] and the increased adhesion of the Lewis antigen to plasma lipoproteins during pregnancy.[5] Both mechanisms result in a significant reduction of expression on the RBC membrane.

Despite the effect of pregnancy on the RBC Lewis phenotype, a clinically significant association between phenotype and PTL or PROM was not supported by the results of our study. These results complement the finding of Lurie et al.,[11] who failed to demonstrate an association between the Lewis phenotype and upper-genital-tract infection in nonpregnant women. In that study, RBC phenotyping for ABO, P, and Lewis antigens was performed in women with at least 3 documented episodes of acute pelvic inflammatory disease. Despite the isolation of *Escherichia coli* as the most commonly (70%) isolated pathogen in their cervical cultures, an increase in Le (a-b-) phenotype was not demonstrated.

The difference in importance of the Lewis phenotype between urinary-tract and genital-tract infection may be secondary to the responsible pathogens. Urinary-tract infections are most frequently caused by pathogenic enteric bacilli for which the Lewis fucosylated carbohydrate chains cause an impediment to attachment. The bacteria associated with pelvic inflammatory disease, PROM, or PTL, in contrast, are varied, including gram-positive bacteria and a high incidence of mycoplasma. The Lewis antigen status should have little influence on colonization and infection with these organisms. The lack of an association of the Lewis phenotype, PROM, PTL, and these organisms implies that the attachment of pathogenic coliforms to the vaginal mucosa is not an important risk component in the pathogenesis of these pregnancy complications. Since infection is an important contributor in only a subset of women with PROM or PTL, the number of women in the study is not sufficient to exclude the possibility of a small effect of Lewis antigen status in these women. We believe, however, that these results preclude the use of Lewis phenotype determination in screening women for an increased risk of these complications.

These results moreover imply that the different frequencies of PTL and PROM seen among racial groups and the tendency for these complications cannot be ascribed to a genetic predisposition for bacterial attachment to the vaginal mucosa. Other mechanisms, including differences in microflora, resistance of cervical mucus to bacterial migration, or differences in host resistance, deserve further study.

### REFERENCES

1. Sheinfeld J, Schaeffer AJ, Cardon-Cardo C, et al.: Association of the Lewis blood-group phenotype with recurrent urinary tract infections in women. N Engl J Med 320:773–777, 1989.
2. Scheinfeld J, Cordon-Cardo C, Fair WR, et al.: Association of type 1 blood group antigens with urinary tract infections in children with genitourinary tract structural abnormalities. J Urol 144:469–473, 1990.
3. Race RR, Sanger R: Blood Groups in Man. 6th ed. Oxford: Blackwell Scientific, pp 323–349, 1975.
4. Schachter H, Michaels MA: A quantitative difference in the activity of blood group A-specific N-acetylgalactosaminyltransferase in serum from A1 and A2 human subjects. Biochem Biophys Res Commun 45:1011–1018, 1971.
5. Hammar L, Mansson S, Rohr T, et al.: Lewis phenotype of erythrocytes and Leb-active glycolipid in serum of pregnant women. Vox Sang 40:27–33, 1981.
6. Navas EL, Venegas MF, Duncan JL, et al.: Blood group antigen expression on vaginal and buccal epithelial cells and mucus in secretor and nonsecretor women. J Urol 149:1492–1498, 1993.
7. Mercer BM, Moretti ML, Prevost RR, Sibai BM: Erythromycin therapy in preterm premature rupture of the membranes: A prospective randomized trial of 220 patients. Am J Obstet Gynecol 166:794–802, 1992.
8. Romero R, Sitori M, Oyarzun E, et al.: Prevalence, microbiology, clinical significance of intraamniotic infection in women with preterm labor and intact membranes. Am J Obstet Gynecol 161:817–824, 1989.
9. Romero R, Avila C, Santhanam U, Sehgal PB: Amniotic fluid interleukin 6 in preterm labor. Association with infection. J Clin Invest 85:1392–1400, 1990.
10. Laham N, Rice GE, Bishop GJ, et al.: Elevated plasma interleukin 6: A biochemical marker of human preterm labor. Gynecol Obstet Invest 36:145–147, 1993.
11. Lurie S, Sigler E, Fenakel K: The ABO, Lewis, or P blood group phenotypes are not associated with recurrent pelvic inflammatory disease. Gynecol Obstet Invest 31: 158–160, 1991.

## References

[PDF_00311] Hammar L., Månsson S., Rohr T., Chester M. A., Ginsburg V., Lundblad A., Zopf D. (1981). Lewis phenotype of erythrocytes and Leb-active glycolipid in serum of pregnant women.. Vox Sang.

[PDF_00329] Laham N., Rice G. E., Bishop G. J., Hansen M. B., Bendtzen K., Brennecke S. P. (1993). Elevated plasma interleukin 6: a biochemical marker of human preterm labour.. Gynecol Obstet Invest.

[PDF_00332] Lurie S., Sigler E., Fenakel K. (1991). The ABO, Lewis or P blood group phenotypes are not associated with recurrent pelvic inflammatory disease.. Gynecol Obstet Invest.

[PDF_00318] Mercer B. M., Moretti M. L., Prevost R. R., Sibai B. M. (1992). Erythromycin therapy in preterm premature rupture of the membranes: a prospective, randomized trial of 220 patients.. Am J Obstet Gynecol.

[PDF_00314] Navas E. L., Venegas M. F., Duncan J. L., Anderson B. E., Chmiel J. S., Schaeffer A. J. (1993). Blood group antigen expression on vaginal and buccal epithelial cells and mucus in secretor and nonsecretor women.. J Urol.

[PDF_00326] Romero R., Avila C., Santhanam U., Sehgal P. B. (1990). Amniotic fluid interleukin 6 in preterm labor. Association with infection.. J Clin Invest.

[PDF_00322] Romero R., Sirtori M., Oyarzun E., Avila C., Mazor M., Callahan R., Sabo V., Athanassiadis A. P., Hobbins J. C. (1989). Infection and labor. V. Prevalence, microbiology, and clinical significance of intraamniotic infection in women with preterm labor and intact membranes.. Am J Obstet Gynecol.

[PDF_00307] Schachter H., Michaels M. A. (1971). A quantitative difference in the activity of blood group A-specific N-acetylgalactosaminyltransferase in serum from A 1 and A 2 human subjects.. Biochem Biophys Res Commun.

[PDF_00301] Sheinfeld J., Cordon-Cardo C., Fair W. R., Wartinger D. D., Rabinowitz R. (1990). Association of type 1 blood group antigens with urinary tract infections in children with genitourinary structural abnormalities.. J Urol.

[PDF_00297] Sheinfeld J., Schaeffer A. J., Cordon-Cardo C., Rogatko A., Fair W. R. (1989). Association of the Lewis blood-group phenotype with recurrent urinary tract infections in women.. N Engl J Med.

